# Multifunctional Arabinoxylan-*functionalized*-Graphene Oxide Based Composite Hydrogel for Skin Tissue Engineering

**DOI:** 10.3389/fbioe.2022.865059

**Published:** 2022-04-27

**Authors:** Muhammad Umar Aslam Khan, Saiful Izwan Abd Razak, Anwarul Hassan, Saima Qureshi, Goran M. Stojanović, Ihsan-Ul-Haq

**Affiliations:** ^1^ BioInspired Device and Tissue Engineering Research Group, School of Biomedical Engineering and Health Sciences, Faculty of Engineering, Universiti Teknologi Malaysia, Johor Bahru, Malaysia; ^2^ Nanosciences and Nanotechnology Department, National Centre for Physics, Quaid-i-Azam University, Islamabad, Pakistan; ^3^ Centre for Advanced Composite Materials, Universiti Teknologi Malaysia, Johor Bahru, Malaysia; ^4^ Department of Mechanical and Industrial Engineering, Qatar University, Doha, Qatar; ^5^ Biomedical Research Center, Qatar University, Doha, Qatar; ^6^ Faculty of Technical Sciences, University of Novi Sad, Novi Sad, Serbia; ^7^ Department of Pharmacy, Quaid-i-Azam University, Islamabad, Pakistan

**Keywords:** antibacterial, anticancer, composite hydrogels, hemocompatibility, skin wound healing, tissue engineering

## Abstract

Wound healing is an important physiological process involving a series of cellular and molecular developments. A multifunctional hydrogel that prevents infection and promotes wound healing has great significance for wound healing applications in biomedical engineering. We have functionalized arabinoxylan and graphene oxide (GO) using the hydrothermal method, through cross-linking GO-arabinoxylan and polyvinyl alcohol (PVA) with tetraethyl orthosilicate (TEOS) to get multifunctional composite hydrogels. These composite hydrogels were characterized by FTIR, SEM, water contact angle, and mechanical testing to determine structural, morphological, wetting, and mechanical behavior, respectively. Swelling and biodegradation were also conducted in different media. The enhanced antibacterial activities were observed against different bacterial strains (*E. coli*, *S. aureus*, and *P. aeruginosa*); anticancer activities and biocompatibility assays were found effective against *U-87* and *MC3T3-E1* cell lines due to the synergic effect of hydrogels. *In vivo* activities were conducted using a mouse full-thickness skin model, and accelerated wound healing was found without any major inflammation within 7 days with improved vascularization. From the results, these composite hydrogels might be potential wound dressing materials for biomedical applications.

## Introduction

The skin, the largest organ of the body and the first line of defense, has different morphological and structural characteristics. The skin serves to protect the internal organs of the body from external influences. The skin cells regenerate wounds due to their natural healing capacity, but it must be done quickly and appropriately for skin wounds (Yadav et al., 2019; Li et al., 2020). Traditional dressings such as bandages, gauze, and sutures have several drawbacks when it comes to wound healing. An ideal wound dressing should promote fast wound healing, remove exudate, provide wetting and gaseous exchange, have adequate mechanical strength, promote growth factors, and protect the wound from microbes and environmental stresses. It must be non-cytotoxic and biodegradable (Liang et al., 2021; Huang et al., 2022). The wound dressing made of cotton gauze does not hydrate the wound and can harm the wound’s regenerative cells when removed. To develop a multifunctional wound dressing, different materials have been used, which are prepared using advanced technologies in various ways (Dong and Guo, 2021). Multifunctional composite hydrogels are ideal wound dressing materials because of their multifunctional properties. The advanced composite hydrogels can control body fluid, moist environment, accelerate healing, reduce inflammation, and hinder bacterial growth by being biocompatible (Yin et al., 2022). Composite hydrogels are multifunctional biomaterials made up of a variety of natural and synthetic polymers as well as fillers. Polyvinyl alcohol (PVA) is a well-known synthetic polymer that has been widely used in wound healing because of its good mechanical properties and biocompatibility. It has poor antimicrobial characteristics, which limits its use as a wound-healing agent (Jia et al., 2018; Kalantari et al., 2020). However, due to its multifunctional behavior, its composite form may achieve desired antibacterial activities and play a significant role in wound healing. Polysaccharides (alginate, arabinoxylan, guar gum, chitosan, and hyaluronic acid) can also be modified into various forms, such as films and foams. The only disadvantage is poor mechanical stability, which can be overcome by incorporating physical or chemical cross-links into hybrid or composite hydrogels (Zhong et al., 2021). Arabinoxylan (ARX) is a biopolymer and a type of polysaccharide and hemicellulose with xylose backbone and arabinose side chains. It is a major fibrous component of several cereal grains and the second most abundant biopolymer in plants after cellulose. Some of the physiological benefits include fecal bulking, cholesterol reduction, glycemic regularity, prebiotic activity, and immune modulation (Khan et al., 2021a; Aslam Khan et al., 2021). Its inherent physicochemical properties, such as water retention, increased viscosity, and gelatinization, make it a versatile material with many different applications in the medical, food, and pharmaceutical industries (Al-Arjan et al., 2020; Khan et al., 2020). Graphene oxide is a reduced form of naturally occurring graphite made up of a single layer of carbon with honeycomb-like networking and sp^2^ hybridization. Due to its biocompatibility and physicochemical properties, it has recently attracted interest in biomedical applications including biosensors, tissue engineering, wound healing, cancer therapy, and drug delivery systems (Khan et al., 2021b; Umar Aslam Khan et al., 2021). It is well known for dissolving kidney stones and has antibacterial and antitussive properties. Pan et al. have reported the synthesis of PVA/GO-based hydrogel for skin healing applications and found enhanced mechanical, self-healing, and super stretchable properties (Pan et al., 2020).

In this study, we present the development of composite hydrogels: the GO-functionalized arabinoxylan was cross-linked with PVA *via* TEOS (cross-linker) using a simple blending method to develop readily available economical composite hydrogel. According to the best of our knowledge, these formulations have never been reported in the open literature. The structural, morphological, mechanical, and wetting analyses were investigated by FTIR, SEM, UTM, and water contact angle, respectively. Swelling and biodegradation were carried out to determine physicochemical properties. The antibacterial and anticancer activities, and biocompatibility of composite hydrogel were found effective against bacteria, *U-87* and *MC3T3-E1*. *In vivo*, a wound-healing mouse model was employed to investigate healing. These composite hydrogels have the potential to heal skin wounds and would be a potential biomaterial for wound dressings.

## Materials and Methods

### Materials

Graphite powder, TEOS, PBS solution, HCl, H_2_SO_4_, absolute ethanol, glacial acetic acid, nutrient broth, and nutrient agar were purchased from Sigma Aldrich, Malaysia. These chemicals were analytically graded and used without any purification.

Preosteoblast (*MC3T3-E1*) cell lines and alpha-MEM (α-MEM) were supplied by ATCC and Hyclone Laboratories Inc., respectively. Fetal bovine serum (FBS) and l-glutamine penicillin/streptomycin were purchased from ThermoFisher Scientific. Male albino mice (BALB/c) weighing 23–25 g (aged 5–7 weeks) were supplied by the National Institute of Health. Approval from the Animal Ethical Committee was obtained to carry out experiments on mice.

### Methods

#### Extraction of Arabinoxylan From Ispaghula Seed Husk

The Ispaghula seed husk was obtained from a local market in Johor Bahru, Malaysia, as a by-product. Iqbal et al. used a well-known method to extract arabinoxylan from the husk of Ispaghula seed (Saghir et al., 2008). The dust and stones from 50 g of seed husk were removed and dispersed in 500 ml of deionized water for 24 h. The pH of the swollen mixture was then raised to 12 by slowly adding a 2.5% solution of sodium hydroxide (NaOH). The husk was separated from the gel by vacuum filtration, and the filtrate was coagulated at pH 3 by adding acetic acid dropwise. The coagulated gel was washed with deionized water to remove acetic acid and freeze-dried to get dried powder of ARX.

#### Hydrogel Fabrication

**SCHEME 1:**
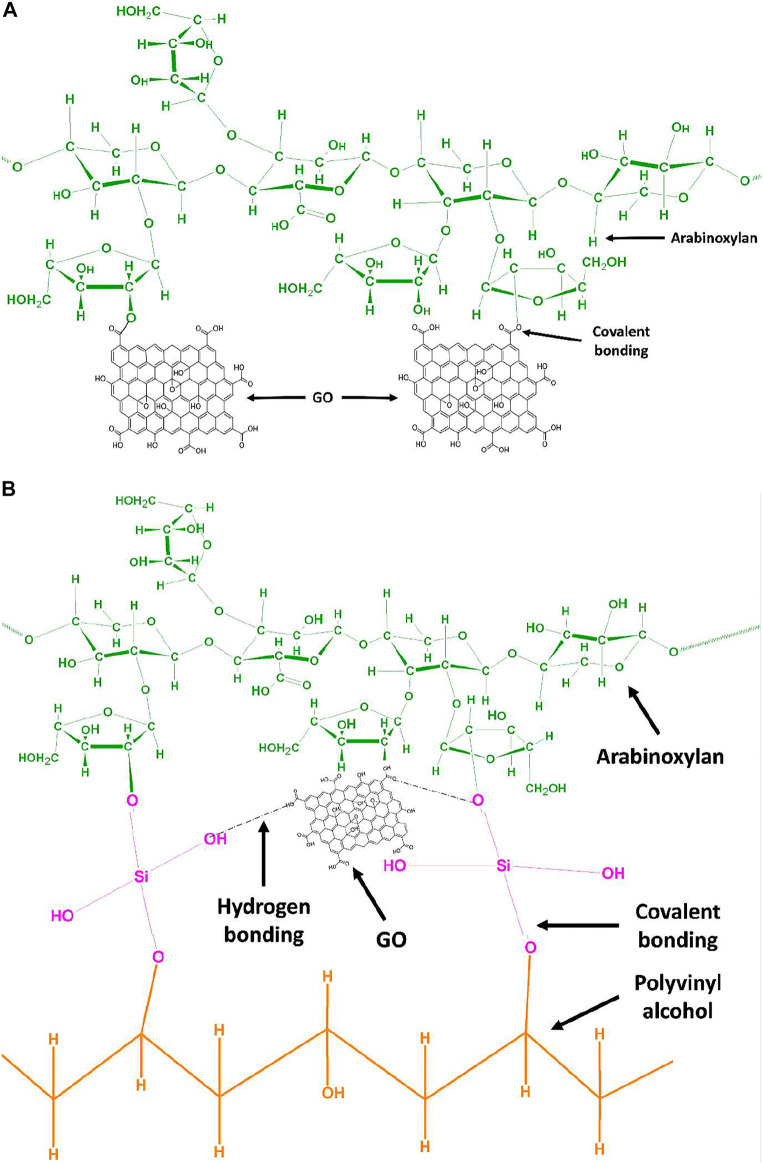
**(A)** Proposed chemical mechanism of GO functionalized arabinoxylan *via* covalent bond interaction by hydrothermal method. **(B)** Proposed chemical mechanism of GO functionalized arabinoxylan and polyvinyl alcohol *via* TEOS cross-linker.

Arabinoxylan was functionalized with GO *via* the hydrothermal method. ARX (2 g) and GO (0.03 mg) were added to the autoclave and kept in an oven at 50°C overnight to get the composite gel, and the proposed schematic is shown in Scheme 1A. The composite gel was dispersed into 25 ml deionized water and stirred with PVA (0.3 g), which is dissolved in 10 ml deionized water at 80°C, for 1 h at 55°C. Different concentrations of TEOS (100, 150, 200, and 250 μL) were dissolved into 5 ml ethanol and added dropwise into the whorl loop of the mixture as a cross-linker. The polymeric mixture was homogenized for 1 h at the same temperature. Then, potassium persulfate (0.2 g) was dissolved in deionized water and added dropwise to the polymeric mixture and allowed to stir for 3 h at 55°C for successful cross-linking. After 3 h, the mixture was poured into Petri plates and dried in the oven at 50°C overnight. Different codes were assigned after different concentrations of TEOS AGP-1 (100 ml), AGP-2 (150 ml), AGP-3 (200 ml), and AGP-4 (250 ml). The proposed reaction cross-linked composite hydrogel is shown in Scheme 1B.

### Characterizations

The structural and functional group identification of composite hydrogels was analyzed by Fourier-transform infrared spectroscopy (Nicolet 5,700, Waltham, MA, United States). The range was 4,000–400 cm^−1^ with 150 scans. The surface morphology of the composite hydrogel was observed by a scanning electron microscope (JEOL-JSM 5410 LV) with an accelerating 10 kV voltage. The well-dried film of composite hydrogels was gold-supported before analysis. The wetting analysis of the composite hydrogel was performed using a water contact angle system (JY-82, Dingsheng, Chengde, China) to investigate the hydrophilicity and hydrophobicity. The mechanical testing of composite hydrogels was conducted by tensile tests, and hydrogel samples were shaped into strips. The tensile testing (length 55 mm, width 15 mm, and thickness 2 mm) and tests were conducted with 10 mm/min speed by ASTM D638 (Standard, 2003).

### Gel Fraction

Weighing small pieces of composite hydrogels was used to conduct the gel fraction analysis. These small composite hydrogel pieces were submerged in deionized water for 12 h at room temperature. These hydrogel samples were then taken out and dried in an oven at 40 °C until they reached a consistent weight (Eq. 3).

$$Gel\;fraction(\%)=\frac{M^{\prime }}{M^{o}},$$ (1)

where ***M*** ^***o***^ is the oven-dried hydrogel weight and $M^{\prime }$ is the initial hydrogel weight.

### Swelling and Biodegradation

At 37°C, the pH sensitivity of composite hydrogels was tested in an aqueous and PBS medium at various pH (1–13). All composite hydrogel samples were cut into squares and properly weighed at 50 mg as an initial weight (*W*_*i*_). After soaking in various conditions, the hydrogel samples were removed, and surface water was removed with a tissue paper before being weighed as the final weight (*W*_*f*_). Eq. 2 was used to calculate the swelling behavior.

$$Swelling(\%)=\frac{W_{f}-W_{i}}{W_{i}}\;\times \mathrm{100}$$ (2)

At pH 7.4, 37°C, in PBS media, *in vitro* biodegradation of well-dried composite hydrogels was investigated. All hydrogel samples were carefully cut into squares with a 50 mg weight and placed in PBS media to see how much weight was lost over time. Eq. 3 was used to calculate the biodegradation of the hydrogel samples.

$$Weight\;loss(\%)=\frac{W_{i}-W_{t}}{W_{i}}\;\times \mathrm{100}$$ (3)

where ***W***_***i***_ = initial weight, ***W***_***f***_ = final weight, and ***W***_***t***_ = weight at time “*t*.”

### *In Vitro* Assay

#### Antibacterial Activities

The antibacterial potential of the composite hydrogels was determined using the disc diffusion method. In the disk diffusion method, nutrient broth, nutrient agar, and all the apparatus used for the assay were first autoclaved to avoid any additional bacterial growth. The bacterial strains were refreshed by using 2 loop-full of bacteria in nutrient broth in test tubes and incubated for 24 h. The nutrient agar was poured into the Petri dish and allowed to settle. The bacterial strains were spread over nutrient agar using a glass rod. Then 75 µL hydrogel samples was placed by micropipette, and the Petri dishes were incubated for 12 h. The antibacterial behavior was recorded in terms of zone inhibition.

#### Hemocompatibility Assay

A hemocompatibility assay was carried out to find the compatibility of the hydrogel polymer with the blood. Hydrogels were assayed at different concentrations ranging from 500 to 100 ug with 5–10% solution of blood cells in PBS. After getting the blood, it was immediately mixed in EDTA solution to prevent the clotting of blood; 500 ul of blood was taken in each Eppendorf and centrifuged at 14,000 rpm for 15 min. Blood cells settled down, and serum was discarded. Blood cells were washed with PBS by adding 1 ml of phosphate buffer saline in this Eppendorf and centrifuged for 15 min. The supernatant was discarded, and the process was repeated 3 times. Isolated red blood cells were then mixed into PBS solution to make a 5% solution of red blood cells. Different concentrations of hydrogel were placed in each Eppendorf followed by the addition of 500 ul of RBC solution. It was then incubated at 37°C for 30 min, then centrifuged again at 14000 rpm for 10 min, 200 ul of supernatant was then collected in 96-well plates, and absorption was measured at 541 nm wavelength.

#### Cytotoxicity and Cell Proliferation

The cytocompatibility and cell proliferation of composite hydrogels with different concentrations have been studied against Uppsala (*U87*) and mouse pre-osteoblast (*MC3T3-E1*) cell lines at different intervals of time (24, 48, and 72 h). The well plates were coated with gelatin (0.1%), which is taken as a positive control. These plates were incubated under standard *in vitro* conditions. Cytotoxicity was conducted using the Neutral Red assay as reported by Repetto (Repetto et al., 2008). The optical density was recorded by absorbance at 540 and 550 nm *via* a microplate reader for *MC3T3-E1* and *U87*, respectively. The cell viability was calculated by Eq. 3.

$$Cell\;vaibility=\frac{OD_{s}}{OD_{c}}\;\times \mathrm{100}$$ (4)

where ***OD***_***s***_ is the sample optical density and ***OD***_***c***_ is the controlled optical density.

### *In Vivo* Wound Contraction

Mice were kept in the animal facility of the Department of Pharmacy, Quaid-i-Azam University, and acclimatized for 1 week in standard environmental conditions. After ensuring that the mice are acclimatized to the environment and are of standard weight, 60 µl of tramadol (25 mg/kg) was orally fed to the mice using a feeding tube and syringe. Hair was removed from the dorsum side of the mice (3 cm down from the neck and between the shoulder blades) by a clipper. Hair removing cream was applied lightly for no longer than 2 min. After 2 min, depilatory cream and fur were removed by using wetted gauze. Mice were placed in a jar for just 5 s, containing cotton wetted with chloroform to give them anesthesia. After cleaning the skin, they were disinfected with 70% alcohol. The skin was lifted from the dorsal side at the midline with the help of the index finger and thumb cranially and caudally. The mice were then placed in a lateral position, and two-layer skin was removed with the help of a sharp biopsy punch to make an excisional wound of 6 mm in diameter. After excision, the mice were placed in a warm area to maintain their body temperature and observed until they recovered from anesthesia. When it gets normal, mice were transferred into their cage. Wound size was measured on alternate days by using a Vernier caliper, and percent wound closure was measured.

### Statistical Analysis

The obtained data were statistically analyzed by statistical software (IBM, SPSS Statistics 21); standard error (S.E.) in the figures was represented as Y-error bars. The two-way ANOVA has been used with *post hoc* multiple comparisons (∗*p* < 0.05, ∗∗*p* < 0.01, and ∗∗∗*p* < 0.001 size of sample, n = 3).

## Results and Discussion

### FTIR Analysis

**FIGURE 1:**
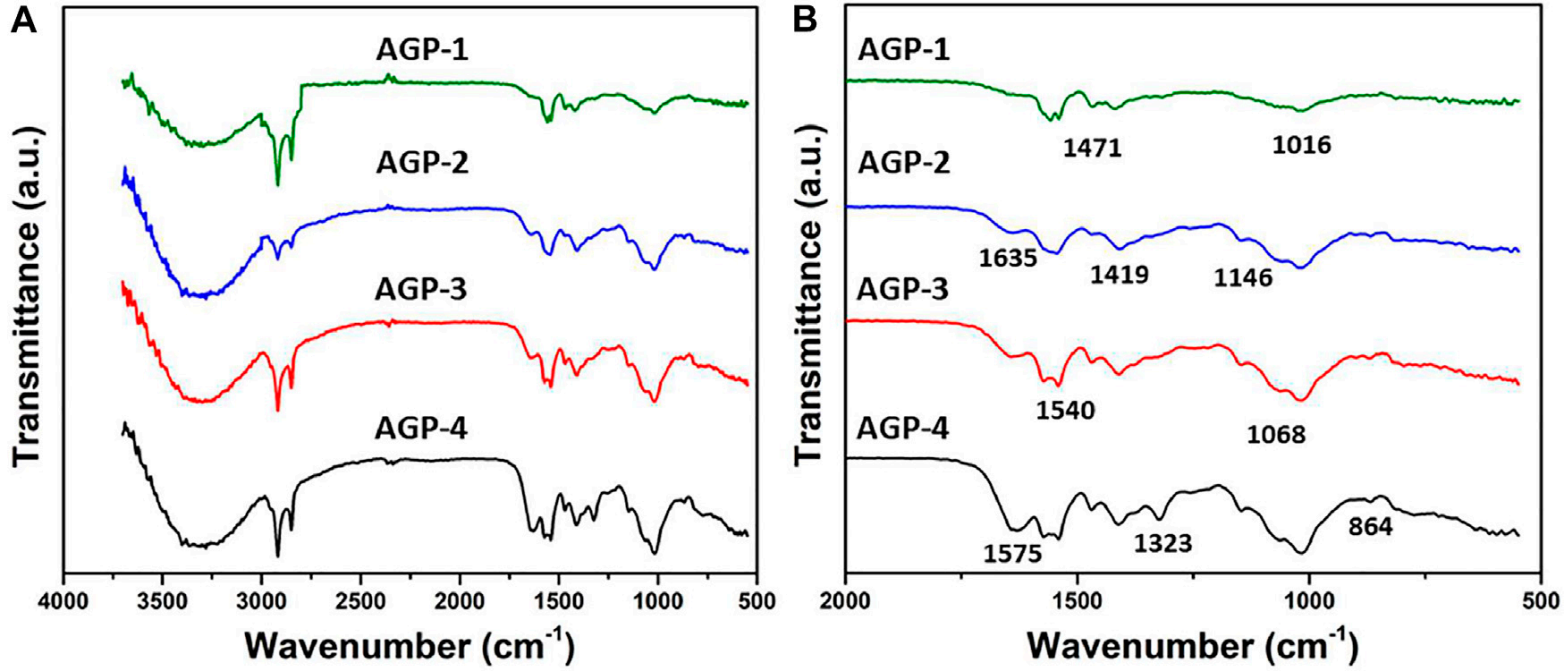
FTIR spectrum of composite hydrogels.

The FTIR spectrum of composite hydrogels with different formulations of composite hydrogels is shown in Figure 1. The broadband peak at 3,750–3,200 cm^−1^ is due to inter/intra hydrogen bonding. It confirms the hydrogen bond of composite hydrogels between arabinoxylan and PVA/GO (Aslam Khan et al., 2021). The stretching bands that were observed at 2,918–2,848 cm^−1^ are attributed to the aliphatic saturated C−H bond. It has different transmittance that is increased with an increased degree of cross-linking. The peak positions at 1,635 and 1,575 cm^−1^ indicate the presence of −C═O stretching band and C═C functional group of graphene oxide. The pyranose ring and saccharine structure peaks were observed at 864 and 1,146 cm^−1^, respectively, which are fundamental peaks of arabinoxylan (Adorinni et al., 2021; Khan et al., 2021c). The stretching band 1,110–1,000 cm^−1^ is due to the presence of TEOS (cross-linker) and it confirms the presence of −Si−O−C and −Si−O−Si functional groups. The increasing transmittance intensities of the stretching band 1,110–1,000 cm^−1^ confirm the increasing amount of TEOS (Fan et al., 2021). Hence, the functional groups and variable transmittance confirm the successful development of a different formulation of composite hydrogels.

### Morphological Analysis

**FIGURE 2:**
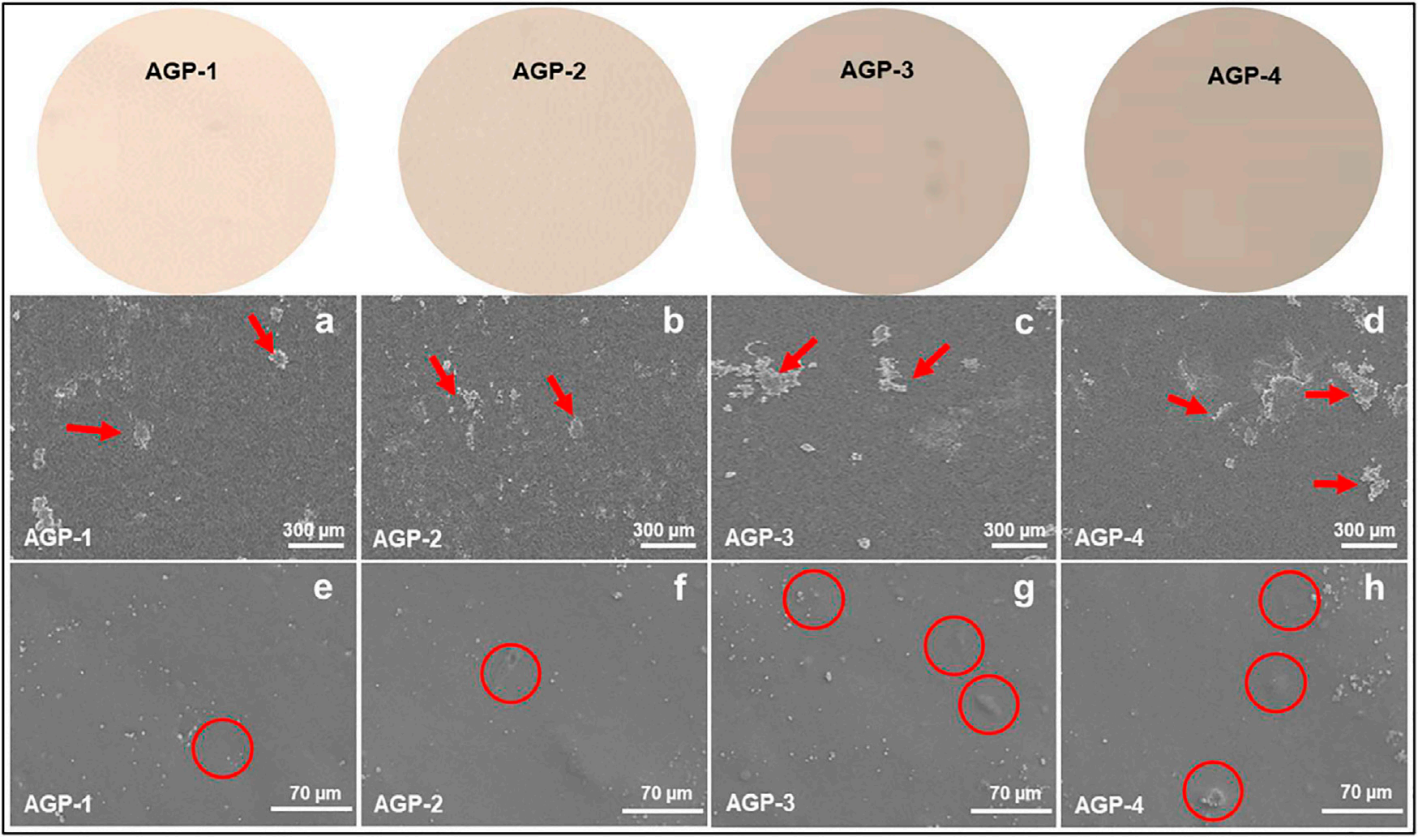
Surface morphology of composite hydrogels at different scales (300 and 70 µm): red arrows indicate GO flakes and red circles indicate embedded GO particles.

The surface morphology of the generated hydrogel was examined using scanning electron microscopy as shown in Figure 2. The morphology of the hydrogels was investigated at various scales. According to the morphological analysis, the hydrogel’s surface morphology is tight, thick, rough, and wavy. It possesses high toughness and dense cross-linking because of the enhanced TEOS amount. It also has a wrinkled, rough lamellar shape, which could be attributed to the presence of GO. This surface is rougher than typical, which helps to increase the surface area. By boosting ion transport and polymer chain mobility, it will improve the self-healing ability of hydrogels (Khan et al., 2021d; Zhao et al., 2022). It was also observed that increasing TEOS amount caused GO to cause more GO clustering at 300 µm as presented with red arrows. However, nano-GO and embedded GO flakes can also be observed in red circles. Hence, the rough surface morphology helps cell adherence and proliferation, while the close packing is formed due to the increasing cross-linker amount that retains its structure after containing substantial biofluids.

### Wetting

**FIGURE 3:**
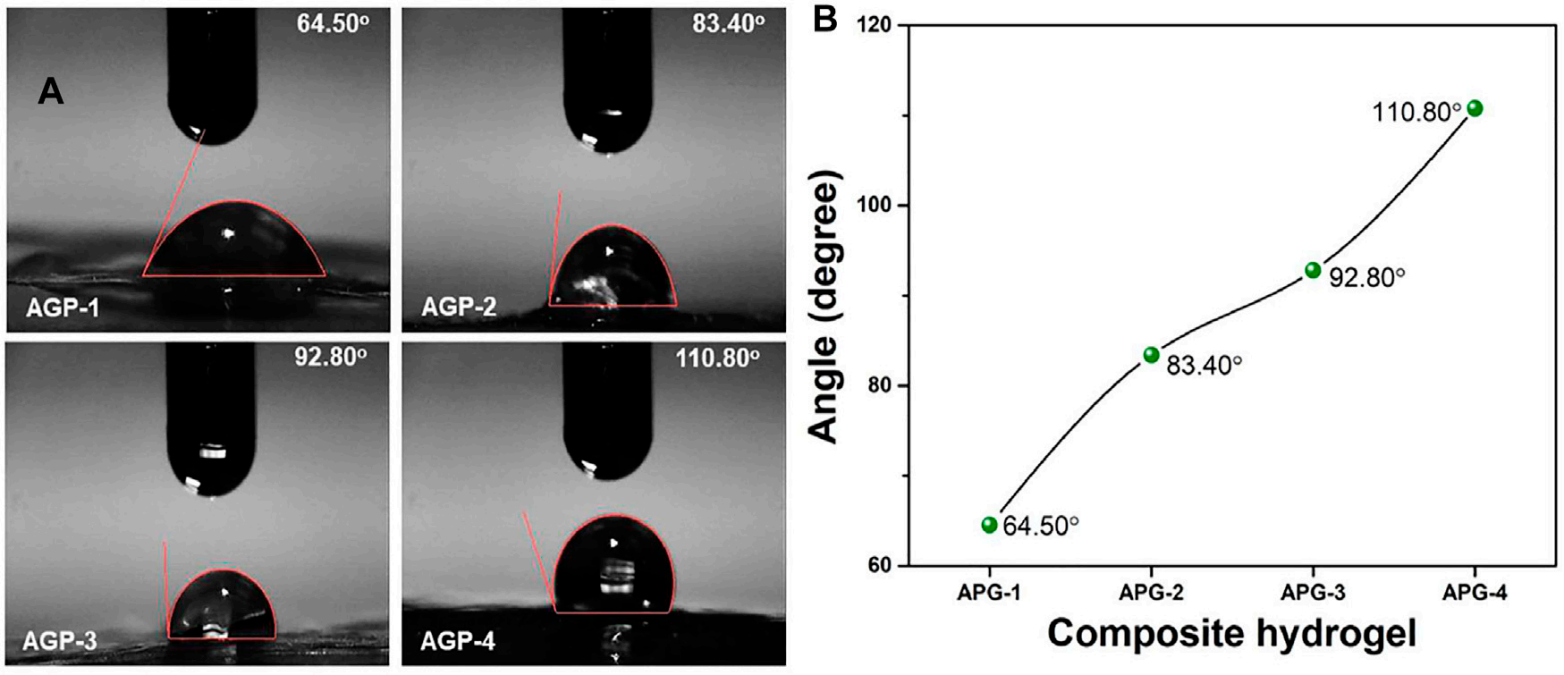
Wetting behavior of composite hydrogels at ambient.

One of the most significant features of all biomaterials is surface wettability because it depicts the true structure and chemical surface properties. Many features of therapeutic drugs, such as biocompatibility, adhesion, lubricity, selective absorption, and controlled release, are determined by wetting (Khan et al., 2021e). Soft hydrogels, which are made primarily of water and a hydrophilic polymeric network, are naturally hydrophilic. Because of their hydrophilic nature, they have a low water contact angle with their surfaces. The wetting behavior changed from hydrophilic to hydrophobic as the cross-linker quantity was increased, owing to increased and close packing that changed surface characteristics. Because of the dense packing and higher cross-linking, there is less hydrogen bonding available to create functional groups (Sala et al., 2021). As a result, the hydrogel sample (AGP-1) was found to have the highest hydrophilicity (64.50°) with minimal cross-linking and the highest hydrophobicity (AGP-4) with maximal cross-linking (110.80°) as presented in Figure 3. The required properties of the hydrogel can be attained by adjusting the cross-linking degree. As a result, we created hydrogels with varying degrees of cross-linking to provide distinct formulations with varying physicochemical properties to address various wound healing applications in various wound environments.

### Mechanical Testing and Gel Fraction

**FIGURE 4:**
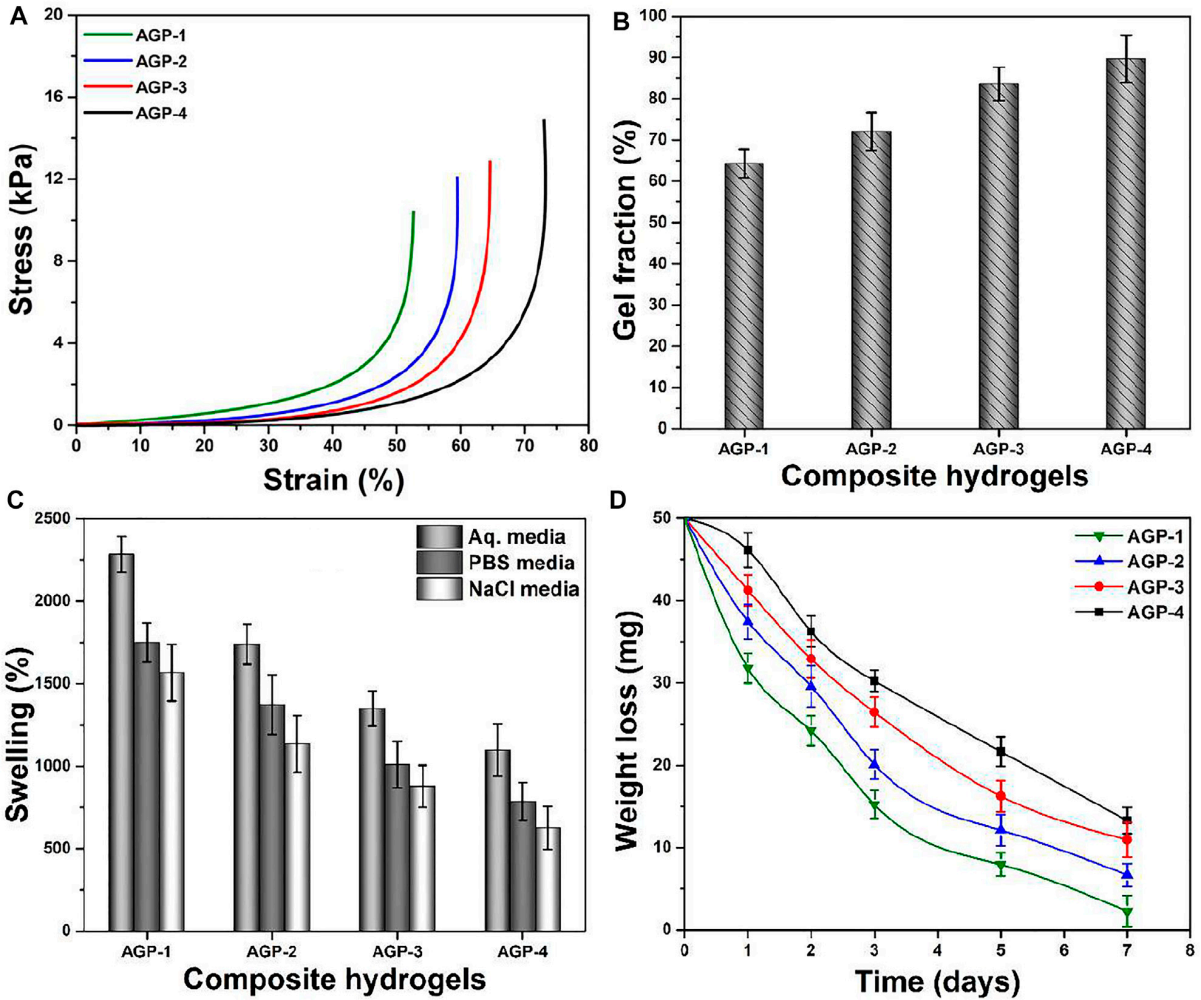
**(A)** Mechanical testing, **(B)** gel fraction (%), **(C)** swelling in different media (aqueous, PBS solution, and NaCl solution), and **(D)** biodegradation in PBS media of composite hydrogels.

The mechanical behavior of composite hydrogel was analyzed by stress–strain curves as shown in Figure 4A; it presents the substantial improvement in mechanical strength by increasing the cross-linking degree (from AGP-1 to AGP-4). The structural and mechanical properties of composite hydrogels were investigated by changing the amount of TEOS to obtain different formulations for wound healing applications. The mechanical behavior will aid in determining structural integrity and cross-linker (TEOS) quantity optimization. After absorbing wound exudate, the composite hydrogel swells during application. An ideal hydrogel material with optimized cross-linking will preserve structural integrity without dissolving or breaking, allowing it to absorb the maximum amount of biofluid and thereby guard against bacterial attack (Lin et al., 2021). The mechanical strength of composite hydrogels can be optimized by cross-linker amount. The elastic modulus of samples AGP-2 to AGP-4 is in the range of the elastic modulus of skin. The cross-linking degree increased by increasing the TEOS amount that will allow consistent adhesion when the composite hydrogel is removed from the skin. The mechanically stable composite hydrogels with bioactive properties could be excellent wound dressings for skin wound healing applications. The cross-linking degree is governed by the gel fraction, and the gel fraction percentage of the composite hydrogels has been calculated as shown in Figure 4B. It can be noticed that hydrogel sample AGP-1 has the lowest gel fraction percentage (64.25%) and hydrogel sample AGP-4 has the highest gel fraction percentage (89.67%). The increasing gel fraction % is attributable to an increase in the amount of cross-linker used to bind the polymeric chains together, or to a higher degree of cross-linking. As a result, increased cross-linking promotes covalent bonding, which improves the gel fraction (%) and may facilitate the physicochemical interaction between the functionalized polymer and the cross-linker.

### Swelling and Biodegradation Analysis

When the hydrogel comes into contact with the wound, it absorbs the exudate and begins to swell, which protects against bacterial infection. As demonstrated in Figure 4C, the swelling hydrogel was performed in different media with a pH of 7.4 at 37°C. Composite hydrogels swelled the most in aqueous media, the least in PBS media, and the least in NaCl (electrolyte) media, according to the findings. The highest swelling in aqueous media may be attributed to the deionized nature of the media, while PBS and NaCl media show less swelling due to ion deposition, which may limit hydrogel porosity and prevent additional solution uptake. It was also discovered that AGP-1 has the most swelling and AGP-4 has the least swelling, which could be attributed to the increasing degree of cross-linking. Because AGP-1 has the least cross-linking, it has more open spaces to retain more solutions than AGP-4, which has fewer broad spaces accessible. After absorbing a significant volume of biofluids, biodegradation is an important occurrence for hydrogels because it allows for the prolonged release of therapeutic agents. As shown in Figure 4D, the biodegradation of composite hydrogel in PBS media at 37°C was determined. The least degraded AGP-4 was discovered, while the most degraded AGP-1 was observed. It could be related to varying levels of cross-linking. As a result, the degree of cross-linking can be used to maximize the biodegradation of composite hydrogels. However, because GO has a large surface area and multiple distinct oxygen-based functional groups, it cannot be ignored in the swelling and biodegradation of composite hydrogels. It conducts cross-linking behavior by interacting with the polymeric matrix of hydrogels in a variety of ways, including weak van Der Waal’s forces of attraction and hydrogen bonding (Khan et al., 2021a; Khan et al., 2021f). Swelling and biodegradation can thus be regulated by adjusting the TEOS concentration to achieve the optimal formulation for the environment because we sometimes require distinct swelling that is not destroyed to keep the wound moist. These composite hydrogel degrees can be used in a variety of wound healing applications in a variety of environments to moisten the wound by absorbing biofluids and speed wound healing. After absorbing biofluid or wound exudate, these composite hydrogels would maintain a moisture environment with controlled biodegradation.

### *In Vitro* Assay

#### Antibacterial Activities

**FIGURE 5:**
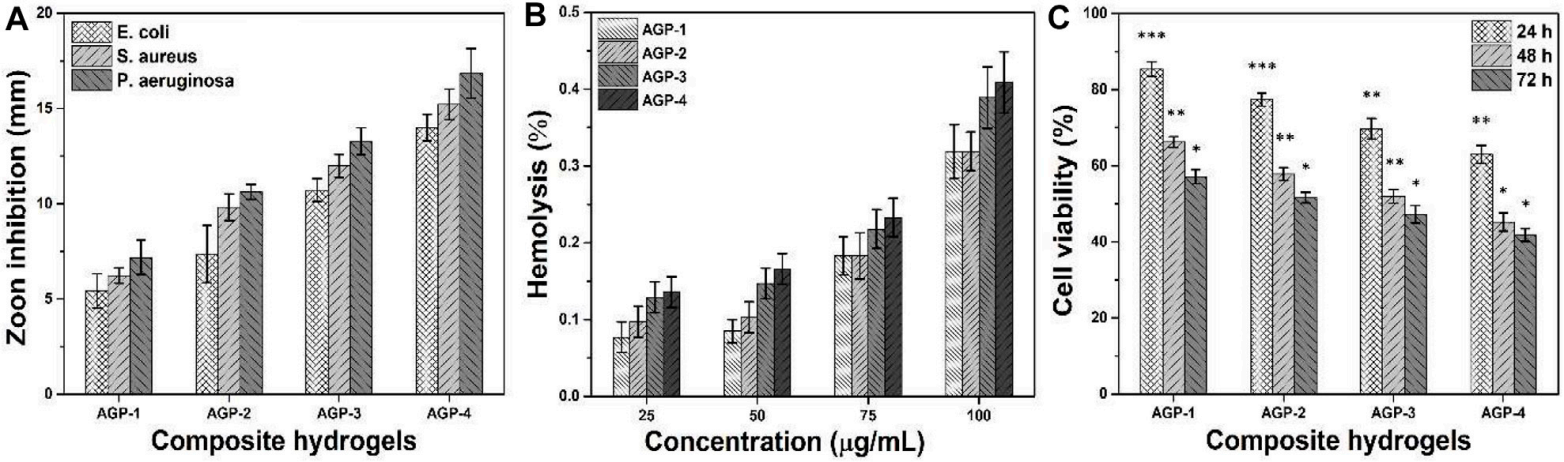
Bioactivities of composite hydrogels were conducted to determine their biological behavior for different activities: **(A)** antibacterial activity against (Gram-positive and Gram-negative) bacterial strains, **(B)** hemocompatibility, and **(C)** anticancer activity. ∗*p* < 0.05, ∗∗*p* < 0.001, and ∗∗*p* < 0.0001.

The antimicrobial properties of any biomaterial are very important in medical applications to provide a biocompatible and protective environment. We have conducted antibacterial activities against Gram-positive and Gram-negative severe infection-causing pathogens, that is, *P. arginase*, *S. aureus*, and *E. coli*, as shown in Figure 5A. The antibacterial activities were presented in terms of zone inhibition. It was observed that increasing cross-linking caused increased antibacterial activities as AGP-1 presented least and AGP-4 maximum. It may be due to the optimized degree of cross-linking that tailored physicochemical properties of the composite hydrogels that interact with pathogens differently. The role of GO cannot be denied; the sharp edges of GO may rupture the bacterial membrane to hinder its activity and replication (Liang et al., 2021; Duan et al., 2022; Kailasa et al., 2022). The polymeric part of the composite hydrogel may interact with the bacterial membrane due to different functional groups and transfer its electrostatic charge. It may interact with bacterial DNA and take over the charge for further replication (Khan et al., 2021f). The synergically composite hydrogels exhibit antibacterial activities for further bacterial replication and growth. Hence, the synergic effect of the composite hydrogel may protect the wound from severe pathogens for proper and quick wound healing.

#### Hemocompatibility Assay

The hemocompatibility of all samples of composite hydrogels has been conducted against healthy human blood, as shown in Figure 5B. A hemocompatibility assay was used to determine the hydrogel polymer’s blood compatibility. Biocompatibility is usually the most significant factor because the materials are used in living beings. The hydrogels aid to speed up the healing process by coming into direct contact with cells and tissues during wound healing. They should not be hemotoxic as a result. According to hemocompatibility data, hydrogel formulations did not tear the membrane of red blood cells, and the cells remained intact. These samples have a hemolysis rate of less than 1%, making them the most blood compatible. It can be seen that these composite hydrogels have different hemocompatibility behavior with different concentrations. Increasing concentration can cause more hemolysis, but the rate is less than 1%. Possibly, the caused hemolysis is due to the GO-sharp edges that may rupture membranes of red blood cells (Duan et al., 2022). Hence, it is confirmed from the results that our composite hydrogels are hemocompatible. These may not cause any serious hemolysis during topical application for skin wound care and treatment.

#### Anticancer Activities

The anticancer activities of composite hydrogels against U87 have been studied at different time intervals (24, 48, and 72 h), as can be seen in Figure 5C. It is worth mentioning that increasing cell incubation time and TEOS amount caused more anticancer activities. Composite polymeric system (ARX-*f*-GO) and TEOS may have a synergetic effect on the U87 cell lines, as GO has sharp edges and ruptures cell membrane to cause cell death. Sample AGP-4 has maximum cell death or less cell viability; however, AGP-1 has more cell viability or less cell death. The polymeric part of the composite hydrogel may take control for further cell proliferation (Ou et al., 2017). Moreover, the longer contact time may also cause cell death to have better anticancer activities (Kumari et al., 2010). It is also observed that initially, the composite hydrogel did not exhibit any prominent anticancer activities. But later, they became more toxic toward U87 cell lines and performed better anticancer activities (Santhosh et al., 2017). This behavior may be due to the optimum amount of polymeric, TEOS, and GO that interact differently with U87 cell lines. These composite hydrogels might be considered potential biomaterials for anticancer applications.

#### Cell Viability and Proliferation

**FIGURE 6:**
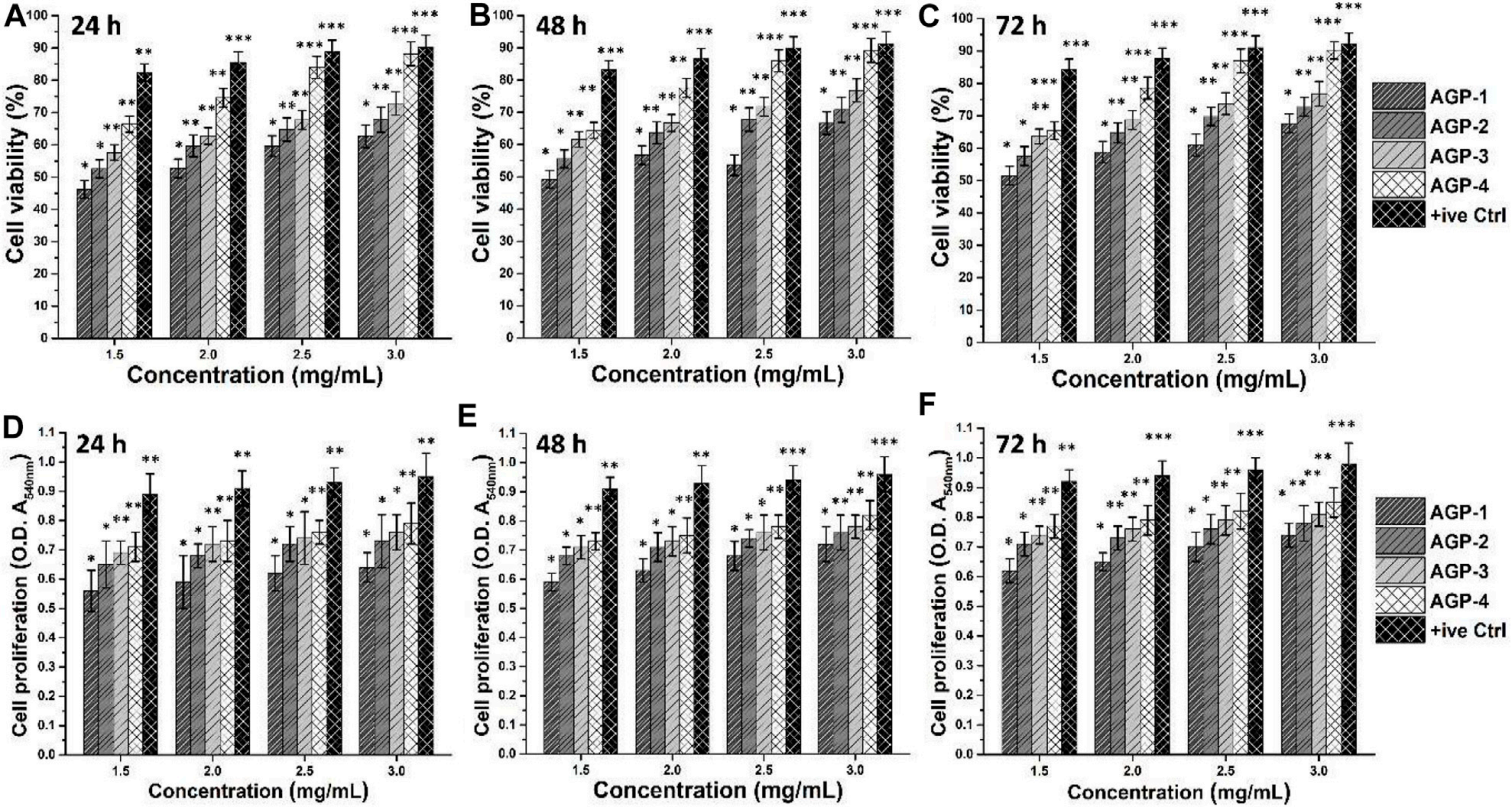
Biocompatibility assays were performed against different concentrations of composite hydrogels to determine the bioactive behavior. The biocompatible behavior was studied by cell viability **(A–C)** and cell proliferation **(D–F)** using *MC3T3-E1* cell lines. Gelatin (0.1%) is taken as a positive control. ∗*p* < 0.05, ∗∗*p* < 0.001, and ∗∗*p* < 0.0001.

Figure 6 shows the vitality and proliferation of *MC3T3-E1* cell lines *in vitro* when exposed to various concentrations of composite hydrogels. Cell survival and proliferation have been demonstrated in these composite hydrogels. Furthermore, it has been demonstrated that when concentration and duration increase, cell viability and proliferation also increase. It could be related to the interaction time of *MC3T3-E1* cell lines with composite hydrogels. It is possible that boosting TEOS and optimizing GO quantity provides the required functionality to assist cell adhesion, which promotes cell viability and proliferation (Hu et al., 2021). The GO has an electroconductive response, and the right amount of GO in a composite hydrogel’s polymeric matrix can produce electroconductive behavior. Since GO contains numerous oxygen-based functional groups, H-bonding aids cell adhesion to hydrogels. GO also has a larger surface area and a variety of functions that may improve cell survival and proliferation by facilitating cell adhesion (Yang et al., 2021). APG-4 >APG-3>APG-2>APG-1 was the order of cell viability and optical density behavior of *MC3T3-E1* cells on the hydrogel scaffold. These findings suggest that raising TEOS and optimizing GO concentrations enhance cell adhesion and proliferation while avoiding cytotoxicity.

### Wound Contraction

**FIGURE 7:**
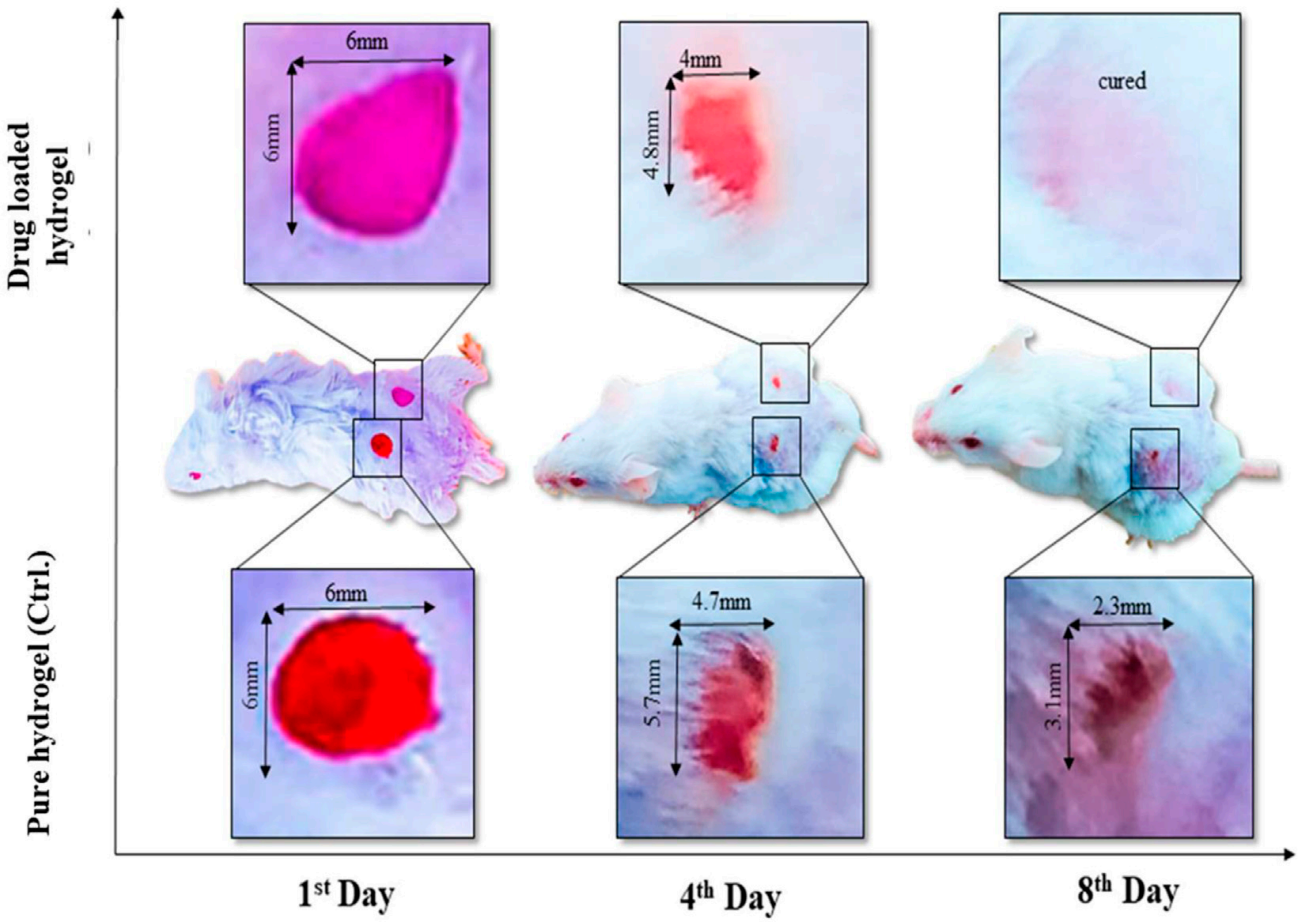
*In vivo* assay (animal model) has presented the wound healing by using a single dose of plan composite hydrogel (control sample) to compare with drug-loaded composite hydrogel.

A wound is a disruption in the continuity of the normal anatomy of the skin. Modern methods of wound healing have advantages over traditional methods. Polysaccharide hydrogel polymers have reduced mechanical strength. Hydrogel polymer composite cross-linked with graphene oxide has tissue regenerative and antimicrobial activity. Hydrogel polymers have their wound healing activity, and when cross-linked, it increases their mechanical strength, and when combined with the drug, it reduces the healing time of the wound. The area of the wound was measured using a scale, and wound closure was noted on days 1, 2, 4, 6, and 8 as shown in Figure 7. Wound contraction of mice showed that hydrogels showed a noteworthy wound healing process, but bergenin-loaded hydrogel had a more significant effect in the wound healing process and helped in the prompt healing process as compared to using hydrogel alone.

## Conclusion

We have reported novel formulations of composite hydrogels with enhanced antibacterial, biodegradable, and bioactivity properties. Arabinoxylan was functionalized with GO *via* the hydrothermal method and cross-linked with PVA using different TEOS amounts to optimize the properties of the hydrogel. FTIR confirms the successful cross-linking, and rough surface morphology was observed by SEM. The wetting behavior was shifted from hydrophilic to hydrophobic by increasing the TEOS amount, while mechanical properties and biodegradation also increased. The multifunctional behavior of GO has tuned the composite hydrogel to have synergic effects on antibacterial, cell viability, and proliferation. It was also found that increasing TEOS amount also enhances cell adherence and proliferation due to increased structural orientation and integrity. The wound healing was observed within 7 days with loading a potential wound-healing drug and plan hydrogel. Amazing wound healing was observed with a single dose of hydrogels loaded on the first day. Therefore, from the results, it is concluded that the composite hydrogel can be a promising biomaterial for healing without any prominent inflammation within a week.

## Data Availability

The raw data supporting the conclusion of this article will be made available by the authors, without undue reservation.
